# Effect of different exercise regimens on LVEF and restenosis incidence in patients after PCI: a network meta-analysis and an overview of systematic reviews

**DOI:** 10.3389/fcvm.2023.1241343

**Published:** 2023-11-14

**Authors:** Hongpeng Li, Li Lu, Zhongyu Han, Zhixiang Liu, Juanhong Pan, Yongsheng Wang, Xiuhua Gao, Yijin Cai, Tianyu Zhao, Qian Nie, Hongcai Zhang, Di Zhang, Song Jin

**Affiliations:** ^1^ School of Medical and Life Sciences, Chengdu University of Traditional Chinese Medicine, Chengdu, China; ^2^ School of Health Preservation and Rehabilitation, Chengdu University of Traditional Chinese Medicine, Chengdu, China; ^3^ Department of Rehabilitation, Hospital of Chengdu University of Traditional Chinese Medicine, Chengdu, China; ^4^ Second Department of Cardiology, Hospital of Chengdu University of Traditional Chinese Medicine, Chengdu, China

**Keywords:** PCI, sports medicine, exercise rehabilitation, meta-analysis, coronary heart

## Abstract

**Objective:**

We aimed to evaluate the effects of different exercise rehabilitation (ER) programs on LVEF and the incidence of restenosis in patients after percutaneous coronary intervention (PCI) through a systematic review and an integrated network meta-analysis (NMA) to provide a reference for the clinical formulation of ER programs for PCI patients.

**Methods:**

Meta-analyses of the effects of different types of ER programs on LVEF and the incidence of reinfarction in post-PCI patients were retrieved from 11 domestic and foreign databases. The methodological and reporting quality of the included systematic reviews were evaluated using the AMSTAR 2 and PRISMA statements. The GRADE scoring system was used to evaluate the quality of evidence found in the studies included in the meta-analysis, and studies with high and intermediate-quality evidence were qualitatively analyzed. Stata software (version 16.0) was used to conduct an integrated NMA of the original RCTs with moderate and low risk of bias.

**Result:**

Sixteen meta-analyses were included in this evaluation. The reporting quality of the included meta-analyses was relatively complete, and the methodological quality was low. Seventy RCTs were included in the NMA. The results showed that all types of rehabilitative exercises were safe and effectively increased LVEF and reduced the incidence of coronary restenosis in patients. The comprehensive exercise program was the most likely to improve LVEF, and the comprehensive exercise program, early exercise program, and high-intensity interval exercise were better than aerobic exercise. Comprehensive exercise programs, early exercise programs, and aerobic exercise reduced the incidence of restenosis in patients. However, Chinese Qigong did not reduce the incidence of restenosis in patients, and there was a risk of bias and inconsistency in the quantitative analysis of restenosis incidence.

**Conclusion:**

Comprehensive exercise programs have the greatest therapeutic significance in improving cardiac output and reducing restenosis rates in post-PCI patients. The early exercise program has great potential but requires kinesiologists to work with physicians to structure the program and strengthen out-of-hospital management. Aerobic exercise has the least therapeutic significance, and Chinese Qigong is suitable for promotion based on its better efficacy than aerobic exercise and may be an alternative to aerobic exercise, but more experimental evidence is needed.

**Systematic Review Registration:**

https://www.crd.york.ac.uk/PROSPERO/, PROSPERO CRD42022374590.

## 1. Background

Coronary heart disease (CHD) is a condition in which atherosclerotic lesions in the coronary arteries cause narrowing or obstruction of the vascular space, leading to myocardial ischemia, hypoxia, or necrosis (1). Percutaneous coronary intervention (PCI) is the most commonly performed procedure in clinical practice to treat CHD, but it cannot eliminate risk factors for CHD or slow down the progression of coronary atherosclerosis (2). Major adverse cardiovascular events (MACEs), such as acute coronary artery occlusion and coronary spasm, often recur after PCI. Meanwhile, psychological problems such as anxiety and depression are also highly prevalent in CHD patients after PCI (3–8). Angina pectoris is the most common symptom, with an incidence of up to 18%–34%, and reinfarction is a primary cause of angina pectoris, cardiac death, and readmission in patients after PCI, with a reinfarction rate of 8.79% 3 years post-surgery (9–12).

As an essential part of modern cardiovascular disease management, cardiac rehabilitation (CR) with exercise rehabilitation (ER) as the core is recommended by the American Heart Association (AHA) and the American College of Cardiology (ACC); it is a reimbursable expense and a Grade A recommendation for treating coronary artery disease and chronic heart failure (13). Current studies have shown that ER can affect microglia-related transcription, regulate sympathetic tone signal transduction, increase heart rate variability, and reduce the probability of malignant arrhythmias. It also inhibits norepinephrine and endothelin-1, protects blood vessels from oxidative stress, improves carbon monoxide bioavailability, and improves endothelial function. At the same time, CR can cause the upregulation of mitochondrial division and change the energy pathway in the organelles to a certain extent, promote fat oxidation over carbohydrate oxidation, improve lipid distribution, improve exercise ability, reduce cardiovascular disease risk factors, and reduce the rehospitalization rate, mortality rate, and incidence of adverse cardiovascular events in patients with CHD (12, 14, 15).

Many systematic reviews and meta-analyses have been published on the effect of ER after PCI. Nevertheless, recommendations for post-PCI early rehabilitation regimens vary among different national or regional guidelines, and some studies have conflicting results. The ACC, American Cardiopulmonary Rehabilitation Association (AACVPR), and AHA support a progression from moderate to vigorous aerobic exercise combined with resistance training. In contrast, according to guidelines upheld throughout Asia, the UK, France, and Japan, lower-intensity aerobic exercise is recommended and resistance training can be reduced or even ignored (16). In China, however, comprehensive exercise is incorporated into traditional Chinese Qigong, such as baduanjin and taijiquan, in some cases (17, 18). As the population of patients undergoing PCI increases worldwide, CR is being increasingly performed; CR is performed in 54.7% of countries in the world (80.7% of European countries); however, it is less frequently performed in developing countries such as China than in developed countries such as European countries and the United States (19). Most community healthcare services are not suited to assist with CR, so it is necessary to synthesize the outcomes of these patients, evaluate the advantages and disadvantages of various exercise programs, and find a suitable exercise mode for the general population and community healthcare service professionals.

Left ventricular ejection fraction (LVEF) refers to the output per beat as a percentage of the end-diastolic volume of the ventricle and is a common clinical indicator of the contractility of the myocardium and output function of the heart. Coronary angiography is the gold standard for diagnosing coronary artery disease, and its review in patients after PCI to determine restenosis occurrence is a direct assessment of prognosis. This study re-evaluated the meta-analysis discussing the efficacy of ER in patients after PCI and performed a mesh meta-analysis involving randomized controlled trials (RCTs) to compare the effect of different exercise regimens on LVEF and restenosis incidence to inform clinicians' decisions to find a suitable exercise mode for the general population and community healthcare services.

## 2. Methods

This scheme was registered in PROSPERO (registration number: CRD42022374590) and is available at https://www.crd.york.ac.uk/PROSPERO/.

### 2.1. Inclusion criteria

Inclusion criteria are based on the PICOS principle.

#### 2.1.1. Population

Patients who had previously undergone PCI, with no restriction on race, age, or sex.

#### 2.1.2. Intervention

ER refers to exercise with specific flow and intensity control performed for medical purposes under the assessment and guidance of physicians, including but not limited to aerobic exercise, resistance training, endurance training, high-intensity interval training, and traditional Chinese exercises, such as Baduanjin.

#### 2.1.3. Comparison

Other non-ER therapies (medication, usual care, physical therapy, blank control, etc.) or ER with a different protocol from the experimental group.

#### 2.1.4. Outcomes

LVEF: Left ventricular ejection fraction was assessed by two-dimensional echocardiography (modified biplanar Simpson method).

Restenosis: Coronary angiography confirmed restenosis ≥50% after the original intervention, including intrastent stenosis.

#### 2.1.5. Study design

Systematic reviews and meta-analyses before 1 April 2023.

#### 2.1.6. Exclusion criteria

① duplicate publications; ② conference studies, reviews, etc.; ③ studies with data that could not be extracted or for which the full text of the literature could not be obtained; ④ studies with incomplete meta-analyses; ⑤ studies with missing critical information; and ⑥ studies in which the evaluation results did not include outcome indicators.

### 2.2. Search strategy

Two researchers independently searched the electronic databases of PubMed, Web of Science, Foreign Medical Literature Retrieval Service (FMRS), Cochrane Library, Embase, Springer, China National Knowledge Infrastructure (CNKI), Wanfang database, Chinese scientific periodical database of Information (VIP), and Chinese Medical Association using a combination of network words and free words. Additionally, references to the included literature and registered PROSPERO were traced. Using PubMed as an example, the retrieval strategy is illustrated in Box 1.

BOX 1 **PubMed search strategy**

#1 percutaneous coronary intervention[MeSH] OR percutaneous coronary intervention[Title/Abstract]

#2 exercise rehabilitation [Title/Abstract] OR Exercise[Title/Abstract] OR Exercise therapy[Title/Abstract]OR training[Title/Abstract]OR Physical training[Title/Abstract]OR kinesis[Title/Abstract]OR motion[Title/Abstract]OR sports medicine[Title/Abstract]OR sport[Title/Abstract]

#3 meta-analysis[MeSH] OR meta-analysis[Publication Type]

#4 meta analysis[Title/Abstract] OR meta analyses[Title/Abstract] OR meta-analysis[Title/Abstract]OR meta-analyses[Title/Abstract] OR data pooling[Title/Abstract]OR data poolings[Title/Abstract] OR clinical study overview[Title/Abstract]OR clinical study overviews[Title/Abstract]

#5#3 OR#4

#6 systematic review[Title/Abstract] OR systematic reviews [Title/Abstract]

#7#5 OR #6

#8#1 AND #2 AND #7

### 2.3. Data extraction and management

Two reviewers independently screened the titles and abstracts of the retrieved results and selected all potentially relevant references according to the search strategy. Bibliographic management was performed using Endnote X9 software.

We used standardized data collection forms to extract the characteristics of the reviews, including the number of original references included, participants, experimental group intervention measures of the control group, and outcome indicators. We contacted the original authors when data needed to be included or information was unclear and analyzed the available data when we could not obtain the required information after sending an email. Disagreements were resolved through discussion or consultation with a third reviewer.

### 2.4. Bias risk (quality) assessment

Two researchers assessed the methodological, reporting, and evidence quality of the included literature, and a third researcher arbitrated conflicts.

#### 2.4.1. Report quality evaluation

The PRISMA statement was used to assess the report quality. There were 27 items in the Preferred Reporting Items for Systematic Reviews and Meta-Analyses statement. A complete report of each item was scored 1 point, a partial report was scored 0.5 points, and an unreported item was scored 0 points. The report was considered somewhat deficient if it scored between 15 and 21 but relatively complete if it scored above 21. According to PRISMA, “items with less than 50% completeness are missing reports” (20, 21).

#### 2.4.2. Methodological quality evaluation

The AMSTAR 2 scale was used to assess the methodological quality of the meta-analyses. The AMSTAR 2 scale contains 16 items, of which Items 2, 4, 7, 9, 11, 13, and 15 are key items. A study with l1 inconsistent non-key item was rated as “high”, a study with >1 inconsistent non-key item was rated as “medium”, a study with 1 inconsistent key item with or without inconsistent non-key items was rated as “low”, and a study with >1 inconsistent key item with or without inconsistent non-key items was rated as “very low”. The AMSTAR 2 score satisfaction and partial satisfaction ≥ 70% can be considered a relatively complete item report (22–24).

### 2.5. Qualitative analysis

Qualitative analysis of the meta-analysis of outcomes and safety, GRADE (25), was used to assess the quality of evidence for different outcome indicators included in the meta-analysis. The degradation factors included study limitations, inconsistency of study results, indirectness or ambiguity (we could not determine whether it was direct evidence), imprecision (insufficient precision or wide confidence interval), and publication bias. The quality of the evidence was graded into four levels: high, moderate, low, and very low, and the conclusions of medium- or high-quality evidence were relatively credible.

### 2.6. Quantitative analysis

The original RCTs included in the meta-analysis for qualitative analysis were analyzed quantitatively, supplemented by the retrieval of updated RCTs. To ensure the quality of the articles, only RCTs with an A or B rating from the Cochrane Collaboration bias risk assessment tool were included. A mesh meta-analysis was performed in Stata 16.0 for the included RCTs using the mesh Meta package and for the three-armed studies, split into two-armed studies with two comparisons for each analysis. Dichotomous variables are expressed herein as odds ratios (ORs) and 95% confidence intervals (CIs), and continuous variables are expressed as standardized mean differences (SMDs) and 95% CIs. Heterogeneity in two-arm comparisons was determined by *I*^2^ and *P* values, with *I*^2^ ≤ 50% and *p* ≥ 0.05 indicating no significant heterogeneity in the included studies. The cumulative surface area under the curve and mean rank were used as indicators to rank the treatment effects of interventions (26–28), and corrected funnel plots were used to detect potential small sample effects or publication bias (29).

## 3. Results

### 3.1. Study screening process and results

**Figure 1:**
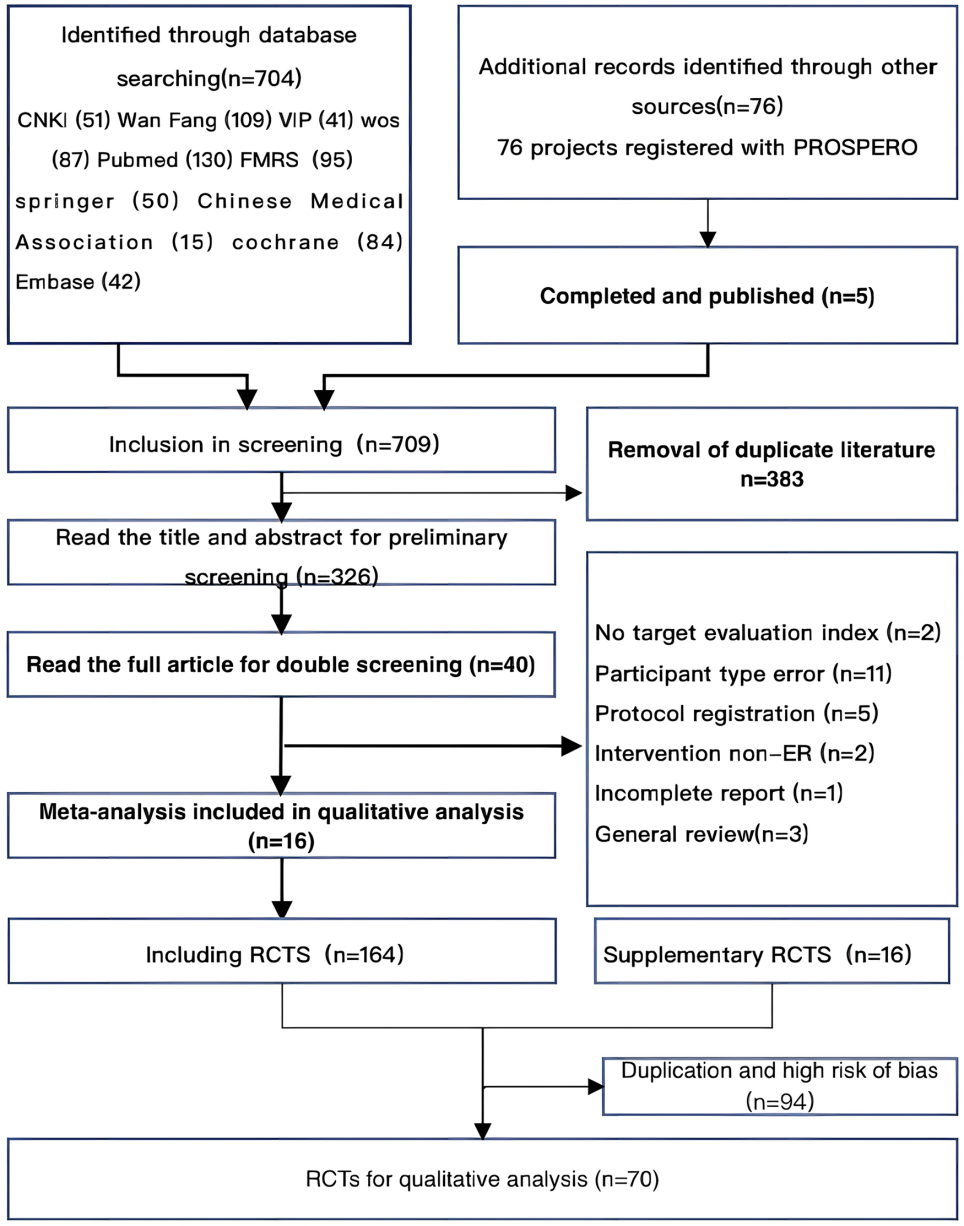
Flow chart for literature screening.

A total of 799 studies and 76 registries were retrieved from 10 databases and PROSPERO. After eliminating duplicates, the titles and abstracts of 326 studies were read for initial screening, and 40 studies were finally selected for reading in full, resulting in the inclusion of 16 meta-analyses for qualitative analysis and 70 RCTs for quantitative analysis. The study retrieval process is illustrated in Figure 1.

### 3.2. Excluded studies

A detailed list of studies and reasons for exclusion can be obtained by contacting the corresponding author of this article.

### 3.3. Basic features of the included literature

Sixteen meta-analyses published between 2013 and 2022 were included in the qualitative analysis, and their main characteristics are shown in Table 1.

(1) Participants: All participants were post-PCI patients included in the meta-analysis.
(2) Intervention measure: The intervention measures in all studies were ER; five studies (34, 36, 39, 43, 45) did not provide details, one study (30) required plans including Baduanjin, and one study (38) required integration with traditional Chinese exercises. One study (42) required the inclusion of high-intensity interval training (HIIT). One study (40) required resistance training as a central intervention.
(3) Control measure: The control measure in one study (42) included moderate-intensity continuous training (MICT) and conventional treatment.
(4) Outcome index: Eleven meta-analyses evaluated LVEF and six evaluated restenosis.
(5) Study type: All literature types were meta-analyses for RCTs; eight studies reported grant support, and all included meta-analyses were correctly assessed for quality. One study (33) used the Jada scale, one (41) used the PEDro scale, one (35) used GRADEpro, and the rest used the Cochrane Collaboration Network bias risk assessment tool.

**Table 1:** Basic features included in the meta-analysis.

Rank	Title	Studies (participants)	Population	Intervention	Comparison	Outcomes	Bias risk assessment tool	AMSTAR 2 ratings	PRISMA scores	Safety evaluation	Funding
1	Xu et al. (30)	8 (696)	Patients undergoing initial PCI	Chinese Qigong	Conventional treatment	①	A	Very low	22.5	Yes	No report
2	Gai et al. (31)	11 (818)	Low-risk patients undergoing PCI	Early ER	Conventional treatment	①	A	Very low	22.5	Yes	No report
3	Gu et al. (32)	8 (586)	AMI patients undergoing initial PCI^h^	Early exercise rehabilitation	Conventional treatment	①	A	Very low	23.5	Yes	YES
4	Meng et al. (33)	18 (1,764)	CHD patients undergoing PCI	Exercise rehabilitation^e^	Conventional treatment	①	C	Very low	24	Yes	YES
5	Ling et al. (34)	9 (944)	Patients undergoing PCI	Exercise rehabilitation	Conventional treatment	②	A	Very low	24.5	Yes	YES
6	Feng et al. (35)	8 (509)	Patients undergoing PCI^g^	Exercise rehabilitation^f^	Conventional treatment	②	D	Very low	21.5	Yes	No report
7	Li et al. (36)	9 (1,600)	Patients undergoing PCI^g^	Exercise rehabilitation	Conventional treatment	②	A	Very low	23.5	Yes	YES
8	Li et al. (37)	16 (1347)	AMI patients undergoing initial PCI	Early exercise rehabilitation	Conventional treatment	①	A	Very low	21.5	Yes	YES
9	Yu et al. (38)	22 (2085)	Patients undergoing PCI	Combined ER^e^	Conventional treatment	①	A	Very low	24	Yes	YES
10	Liu et al. (39)	11 (1227)	CHD patients treated by PCI G	Exercise rehabilitation	Conventional treatment	①②	A	Very low	22.5	Yes	YES
11	Qiu et al. (40)	7 (776)	CHD Patients undergoing initial PCI	Resistance movement	Conventional treatment	①	A	Very low	24	Yes	YES
12	Fu et al. (41)	7 (441)	Patients undergoing PCI^g^	Rehabilitation exercise^f^	Conventional treatment	②	B	Very low	24.5	Yes	YES
13	Zhang et al. (42)	6 (247)	CHD patients undergoing PCI	HIIT	Conventional treatment or MICT	①	A	Very low	24	Yes	YES
14	Yang et al. (43)	6 (682)	Patients undergoing PCI	Exercise rehabilitation	Conventional treatment	②	A	Very low	23.5	Yes	No report
15	Zhang et al. (44)	10 (1274)	CHD patients treated by PCI G	Exercise rehabilitation	Conventional treatment	①	A	Very low	23	Yes	No report
16	Wang et al. (45)	8 (582)	AMI patients treated by PCI	Exercise rehabilitation	Conventional treatment	①	A	Very low	24.5	Yes	No funding

Combined exercise rehabilitation: exercise rehabilitation consisting of integrated Chinese and western medicine, including aerobic exercise impedance, Tai Chi, Baduanjin, etc.; early exercise rehabilitation: exercise rehabilitation starting within 3 days of surgery; HIIT, high-intensity interval exercise; MICT, moderate-intensity continuous training; AMI, acute myocardial infarction; CHD, coronary heart disease; PCI, percutaneous coronary intervention; ①left ventricular ejection fraction (LVEF) ②: restenosis.

^a^ The Cochrane collaboration's bias risk assessment tool.

^b^ PEDro Scale.

^c^ Jadad Scale.

^d^ GRADEpro.

^e^ The duration of intervention was more than 3 months.

^f^ The intensity standard is to achieve 75% of the maximum heart rate.

^g^ Exhibited coronary stenosis of <50% after PCI.

^h^ Radial artery surgery.

### 3.4. Methodological quality evaluation

The methodological quality of 16 meta-analyses was “very low”. There were nine entries in which AMSTAR 2 scores met or partially met ≥70%. Among the seven key items in the AMSTAR 2 quality evaluation, Items 2 and 7 had significant deficiencies, as shown in Table 2.

**Table 2:** Methodological quality of the included meta-analyses evaluated by the AMSTAR 2.

Rank	Title	Item 1	Item 2	Item 3	Item 4	Item 5	Item 6	Item 7	Item 8	Item 9	Item 10	Item 11	Item 12	Item 13	Item 14	Item 15	Item 16	Ratings
1	Xu et al. (30)	Y	N	N	PY	Y	Y	N	Y	PY	N	Y	N	N	N	Y	N	Very low
2	Gai et al. (31)	Y	N	Y	Y	Y	Y	N	Y	Y	N	Y	Y	Y	Y	Y	N	Very low
3	Gu et al. (32)	Y	N	N	Y	Y	Y	N	Y	Y	N	Y	Y	Y	Y	N	Y	Very low
4	Meng et al. (33)	Y	N	N	Y	Y	Y	N	Y	N	N	Y	Y	Y	Y	Y	Y	Very low
5	Ling et al. (34)	Y	N	Y	Y	Y	Y	N	Y	Y	Y	Y	Y	Y	Y	Y	Y	Very low
6	Feng et al. (35)	Y	N	N	Y	Y	Y	N	Y	Y	N	Y	Y	Y	Y	Y	N	Very low
7	Li et al. (36)	Y	N	N	Y	Y	Y	N	Y	PY	N	Y	N	N	N	N	Y	Very low
8	Li et al. (37)	Y	N	N	Y	Y	Y	N	Y	PY	N	Y	N	N	N	N	Y	Very low
9	Yu et al. (38)	Y	N	N	PY	Y	Y	N	Y	PY	N	Y	N	N	Y	Y	Y	Very low
10	Liu et al. (39)	Y	N	N	PY	Y	Y	N	N	Y	N	Y	N	N	N	Y	Y	Very low
11	Qiu et al. (40)	Y	N	N	Y	Y	Y	N	Y	Y	N	Y	Y	Y	Y	Y	Y	Very low
12	Fu et al. (41)	Y	N	N	Y	Y	Y	N	Y	Y	N	Y	Y	Y	Y	Y	Y	Very low
13	Zhang et al. (42)	Y	Y	Y	Y	Y	Y	N	Y	Y	Y	Y	N	Y	N	N	Y	Very low
14	Yang et al. (43)	Y	N	N	PY	Y	Y	N	Y	Y	N	Y	N	Y	Y	N	N	Very low
15	Zhang et al. (44)	Y	N	Y	PY	Y	Y	N	Y	Y	N	Y	Y	Y	Y	Y	N	Very low
16	Wang et al. (45)	Y	N	N	PY	Y	Y	N	Y	Y	N	Y	N	Y	Y	Y	Y	Very low

# is a key entry, Y is yes, N is no, and PY is partial yes. ≤1 noncritical entry does not match as “high”, >1 noncritical entry does not match as “medium”, if 1 critical entry does not match with or without non-critical entry does not match as “low”, >1 critical entry does not match with or without noncritical Very low” if 1 key entry is not met with or without a non-key entry. Entry 1: Do the study questions and inclusion criteria cover the components of the PICO? Entry 2: Is there a pre-published protocol? Study and protocol: Is there significant bias? Does the review contain a clear statement that the study protocol was established before the study was conducted and that there was no significant bias between the study and the protocol? Entry 3: Did the authors explain the type of study design included? Entry 4: Was a comprehensive literature search strategy used? Entry 5: Was duplicate study screening conducted? Entry 6: Were duplicate data extracts performed? Entry 7: Is a list of excluded studies provided, with reasons for exclusion? Entry 8: Was a detailed description of the included studies provided? Entry 9: Was the risk of bias for each included study assessed using a reasonable tool? Entry 10: Are the sources of funding for the included studies reported? Entry 11: If a meta-analysis was conducted, were the results statistically combined using appropriate methods? Entry 12: If a meta-analysis was conducted, is the effect of risk of bias described in the results? Entry 13: If a meta-analysis was performed, is the impact of the risk of bias described in the discussion? Entry 14: Is heterogeneity justified in the discussion? Entry 15: If a quantitative analysis was performed, was publication bias adequately investigated and its possible impact discussed? Entry 16: Are any potential sources of conflict of interest reported?

### 3.5. Reporting quality assessment

The PRISMA scores of the studies ranged from 21.5 to 24.5 (Table 1). One study with severe quality defects (score 14.5) was excluded. The other studies were relatively complete. The PRISMA scores are listed in Table 3. Exceeding half studies were evaluated as “not satisfied” in item “scheme” and “registration”. Exceeding half studies were evaluated as “partially satisfied” in item “introduction rationale”, “eligibility criteria”, “search”, and “limitations”.

**Table 3:** PRISMA-reported scores included in the meta-analysis.

Item	Xu et al. (30)	Gai et al. (31)	Gu et al. (32)	Men et al. (33)	Ling et al. (34)	Feng et al. (35)	Li et al. (36)	Li et al. (37)	Yu et al. (38)	Liu et al. (39)	Qiu et al. (40)	Fu et al. (41)	Zhang et al. (42)	Yang et al. (43)	Zhang et al. (44)	Wang et al. (45)
1. Title	1	1	1	1	1	1	1	1	1	1	1	1	1	1	1	1
2. Abstract	1	1	1	1	1	1	1	1	1	1	1	1	1	1	1	1
3. Introduction Rationale	1	0.5	1	0.5	1	0.5	1	0.5	0.5	0.5	1	0.5	0.5	0.5	0.5	0.5
4. Objectives	1	1	1	1	1	1	1	1	1	1	1	1	1	1	0.5	1
5. Protocol and registration	0	0	0	0	0	0	0	0	0	0	0	0.5	0	0	0	0
6. Eligibility criteria	1	0.5	1	1	1	0.5	0.5	0.5	0.5	0.5	0.5	0.5	1	1	0.5	0.5
7. Information sources	1	1	1	1	1	1	1	1	1	1	1	1	1	1	1	1
8. Search	0.5	0.5	0.5	0.5	0.5	0.5	0.5	0.5	0.5	0.5	0.5	0.5	0.5	0.5	0.5	1
9. Study selection	1	1	1	1	1	0.5	1	1	1	1	1	1	1	1	1	1
10. Data collection	1	1	1	1	1	1	1	1	1	1	1	1	1	1	1	1
11. Data items	0.5	0.5	1	1	0.5	0.5	1	0.5	0.5	0.5	1	1	1	1	1	1
12. Risk of bias in the individual studies	1	1	1	1	1	1	1	1	1	1	1	1	1	1	1	1
13. Summary measures	1	1	1	1	1	1	1	1	1	1	1	1	1	1	1	1
14. Synthesis of the results	1	1	1	1	1	1	1	1	1	1	1	1	1	1	1	1
15. Risk of bias across studies	1	1	0.5	1	1	1	1	0.5	1	1	1	1	1	1	1	1
16. Additional analyses	1	1	1	1	1	1	1	1	1	1	1	1	1	1	1	1
17. Results and study selection	0.5	1	1	0	1	0	1	1	1	1	1	1	1	1	1	1
18. Study characteristics	0.5	1	0.5	1	1	1	1	0.5	1	1	1	0.5	1	1	1	1
19. Risk of bias within studies	1	0.5	0.5	1	1	1	1	1	1	1	1	1	1	1	1	1
20. Results of individual studies	1	1	1	1	1	1	1	1	1	1	1	1	1	1	1	1
21. Synthesis of results	1	1	1	1	1	1	1	1	1	1	1	1	1	1	1	1
22. Risk of bias across studies 22 Risk	0.5	1	1	1	0.5	1	0.5	0.5	1	1	0.5	1	0.5	1	1	1
23. Additional analysis	1	1	1	1	1	1	1	1	1	0.5	1	1	1	1	1	1
24. Summary of evidence	1	1	1	1	1	1	1	1	1	1	1	1	1	1	1	1
25. Limitations	1	1	0.5	1	1	1	0	0	1	1	0.5	1	0.5	0.5	1	0.5
26. Conclusions	1	1	1	1	1	1	1	1	1	1	1	1	1	1	1	1
27. Funding	0	0	1	1	1	0	1	1	1	0	1	1	1	0	0	1
Total points	22.5	22.5	23.5	24	24.5	21.5	23.5	21.5	24	22.5	24	24.5	24	23.5	23	24.5

Each item scores 1 point for a complete report and 0.5 points for a partial report, with a total score greater than 21 being a complete report, 21–15 being a relatively complete report, and less than 15 being an incomplete report.

### 3.6. Qualitative analysis

(1) LVEF: Twelve meta-analyses (30–33, 37–40, 42–45) assessed LVEF and the GRADE rating of these outcomes were 5/12 (31, 32, 40, 43, 44) considered high, 4/12 (30, 33, 37, 42) considered moderate, and 3/12 considered low (30, 31, 45). The high and moderate evidence outcomes all showed that ER could improve the LVEF in patients after PCI, except one (34), which was meaningless (*p* = 0.23) when comparing HIIT with MICT. The combined effect size of early ER as an intervention in two pieces of evidence (34, 37) (MD = 5.57, MD = 5.18) was significantly better than that of the others. The results of Baduanjin (25) (MD = 3.85) were similar to those of the others (Table 4).
(2) Restenosis: Six meta-analyses (30–36, 39, 41, 43) evaluated restenosis recurrence rates, with 2/6 (35, 41) showing high evidence quality outcomes, 4/6 (34, 36, 39, 43) showing moderate evidence quality outcomes, and 1/6 (34) showing low evidence quality outcomes. All high- and moderate-quality evidence outcomes suggested that ER reduces the incidence of restenosis after PCI, but one study (43) was not significant (*p* = 0.1) (Table 4).
(3) Safety: All 16 meta-analyses reported that ER was safe (Table 1).

**Table 4:** GRADE quality of evidence grading for inclusion of meta-analysis outcomes.

Study	Conclusion	Number (patients)	Combined effect size (95% CI)	P	*I* ^2^	Publication bias	Inconsistency	Indirectness	Imprecision	Risk of bias	Quality of evidence
Yang et al. (43)	ER was superior to conventional treatment in improving LVEF	5 (735)	MD: 2.82 (1.50, 4.14)	<0.0001	0.0001	0	0	0	0	0	High
Zhang et al. (44)	ER was superior to conventional treatment in improving LVEF	5 (735)	MD: 2.82 (1.50, 4.14)	<0.0001	0.0001	0	0	0	0	0	High
Qiu et al. (40)	Resistance exercise was superior to conventional treatment in improving LVEF	3 (186)	MD: 4.06 (0.72,7.40)	0.02	0.38	0	0	0	0	0	High
Gu et al. (32)	Early ER was superior to conventional treatment in improving LVEF	4 (324)	MD: 5.75 (4.52,6.99)	<0.0001	0	0	0	0	0	0	High
Gai et al. (31)	Early ER was superior to conventional treatment in improving LVEF	8 (570)	MD: 3.60 (2.73,4.47)	<0.0001	0.18	0	0	0	0	0	High
Xu et al. (30)	Chinese Qigong improves LVEF better than conventional treatment	2 (90)	MD: 3.85 (2.41,5.29)	<0.0001	0.12	0	0	0	0	−1④	Medium
Men et al. (33)	ER was superior to conventional treatment in improving LVEF	11 (1126)	MD: 3. 53 (1. 98, 5. 07)	<0.01	0.71	0	−1②	0	0	0	Medium
Li et al. (37)	Early ER was superior to conventional treatment in improving LVEF	7 (565)	MD: 5. 18 (4. 13,6. 22)	<0.001	0.47	0	−1②	0	0	0	Medium
Zhang et al. (42)	ER was superior to conventional treatment in improving LVEF	2 (83)	SMD: 0.28 (−0.16, 0.63)	0.23	0.3	0	0	0	−1③	0	Medium
Yu et al. (38)	ER was superior to conventional treatment in improving LVEF	12 (901)	MD: 3. 62 (2. 06, 5. 18)	<0.00001	0.63	0	−1②	0	0	−1④	Low
		10 (1184)	MD: 5. 02 (2. 45, 7. 59)	0.0001	0.95	0	−1②	0	0	−1④	Low
Liu et al. (39)	ER was superior to conventional treatment in improving LVEF	5 (639)	MD: 3. 65 (2. 00,5. 30)	<0.0001	0.76	0	−1②	0	0	−1④	Low
	ER was superior to conventional treatment in reducing restenosis.	6 (837)	OR: 0. 36 (0. 21,0. 61)	0.0002	0.3	0	0	0	0	−1④	Medium
Wang et al. (45)	ER was superior to conventional treatment in improving LVEF	8 (582)	SMD: 1.33 (0.43, 2.23)	0.004	0.95	0	−1②	0	0	−1④	Low
Lin et al. (34)	ER was superior to conventional treatment in reducing restenosis.	5 (411)	OR: 0. 39 (0. 23,0.67)	<0.0001	0.46	−1①	0	0	0	−1④	Low
		4 (533)	OR: 0. 32 (0. 21, 0.51)	<0.0001	0.16	−1①	0	0	0	0	Medium
Feng et al. (35)	ER was superior to conventional treatment in reducing restenosis.	8 (509)	OR: 0.40 (0.25, 0.64)	<0.0001	0	0	0	0	0	0	High
Li et al. (36)	Early ER was superior to conventional treatment in reducing restenosis.	3 (687)	OR: 0.54 (0.33,0.87)	0.01	0.01	0	−1②	0	0	0	Medium
Fu et al. (41)	ER was superior to conventional treatment in reducing restenosis.	7 (441)	OR: 0.46 (0.26,0.82)	<0.00001	0	−1①	0	0	0	0	High
Yang et al. (43)	ER was superior to conventional treatment in reducing restenosis.	3 (479)	OR: 0.46 (0.19,1.16)	0.1	0.34	0	0	0	−1③	0	Medium

0, no downgrade; −1, one downgrade; −2, two downgrades; ① the inclusion of studies with limitations in randomization, allocation concealment and blinding, risk of bias, one downgrade; ② indicates high heterogeneity because of poor overlap of confidence intervals or large *I*^2^ values for combined results, moderate heterogeneity (*I*^2 ^> 40%), one downgrade; high heterogeneity (*I*^2^ > 75%), two downgrades; ③small number of included studies, small sample size, wide 95% confidence intervals, potential for publication bias, one downgrade; ④ few included studies or asymmetric funnel plots, one downgrade.

### 3.7. Qualitative analysis

#### 3.7.1. Incorporation of RCT essential features

A total of 70 compliant RCTs were included, with the basic characteristics shown in the Supplementary Table S1. The years of publication were between 1992 and 2023. The participants were all post-PCI patients, and the mean age was clustered between 50 and 75 years, with a high proportion of male participants. Fifty-seven of the RCTs evaluated LVEF, and 14 evaluated restenosis and the included RCT interventions. The different exercise programs involved in the interventions are defined in Table 5.

**Table 5:** Different ER schemes and definitions.

Rank	Scheme	Definition
1	EEP	an in-hospital ER program that begins 1–3 days after PCI and includes an in-hospital exercise prescription and an out-of-hospital maintenance training program. The in-hospital program gradually transitions from passive exercise and simple bedside activities to resistance and aerobic training at a certain intensity, while the maximum intensity of the in-hospital program is maintained outside the hospital.
2	AE	aerobic exercise that does not rely on equipment or a treadmill and does not require any intensity, including walking, jogging with no intensity requirement, or unsupervised exercise.
3	Qigong	traditional Chinese medicine Qigong exercise methods, such as Baduanjin, Taijiquan, Wuqingxi, and Liuzijue.
4	RE	refers to the use of elastic bands, dumbbells, barbells, and other equipment with a certain intensity, planned active exercise against resistance, and self-weight resistance exercise, such as pull-ups and deep squats, continuous training that is maintained for a certain period at an intensity of 60%–70% of the peak heart rate, including power cycling, running, or other training at the required intensity, with sufficient rest time. No specific training intensity is needed.
5	MICT	continuous training that is maintained for a certain period at an intensity of 60–70% of the peak heart rate, including power cycling, running, or other training at the required intensity
6	HIIT	alternating bursts of high-intensity exercise to achieve ing, or other training at the required intensity, with sufficient rest time. No specific training intensity is needed.xercise, su min, generally relying on equipment such as power bikes and treadmills and supervised by a rehabilitation center or hospital.
7	CEP	a comprehensive training program that includes aerobic training, resistance training, flexibility training, and a plan for the intensity and duration of the training, usually prescribed in the form of an exercise prescription, which is adjusted according to the patient's condition during the training process, mostly in a rehabilitation center or hospital.
8	RT	Include normal daily physical activity, such as various medications and routine care.

ER, exercise rehabilitation; EEP, early exercise program; AE, aerobic exercise; qigong, Chinese medicine qigong; RE, resistance exercise; MICT, medium intensity continuous training; HIIT, high-intensity interval exercise; CEP, comprehensive exercise program; RT, routine treatment; HRR, heart rate reserve.

### 3.8. Assessment of bias in included RCTs

**Figure 2:**
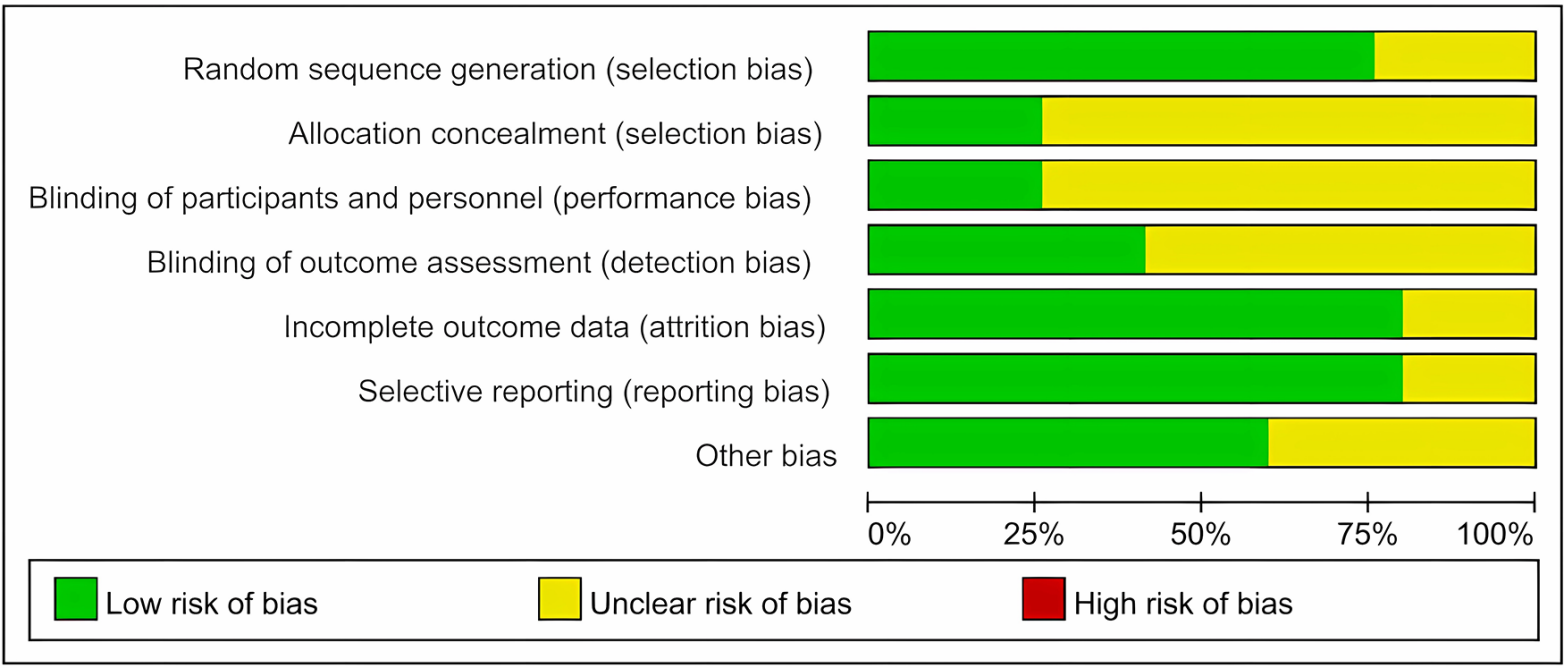
Risk of bias for inclusion in RCTs.

The included RCTs were all at risk of bias assessment level B or above, and RCTs with a high risk of bias were excluded. The main reasons for bias were that most RCTs mentioned allocation concealment methods and blinding but did not describe them, some RCTs did not report self-help sources, and other biases could not be determined (Figure 2).

### 3.9. Net meta-analysis

#### 3.9.1. Inconsistency test

(1) LVEF: The result of the global inconsistency test for LVEF was *p* = 0.3848 (>0.05), with no significant heterogeneity.
(2) Restenosis: The result of the global inconsistency test for restenosis was *p* = 0.038 (<0.05), suggesting heterogeneity.

#### 3.9.2. Network evidence diagram

**Figure 3:**
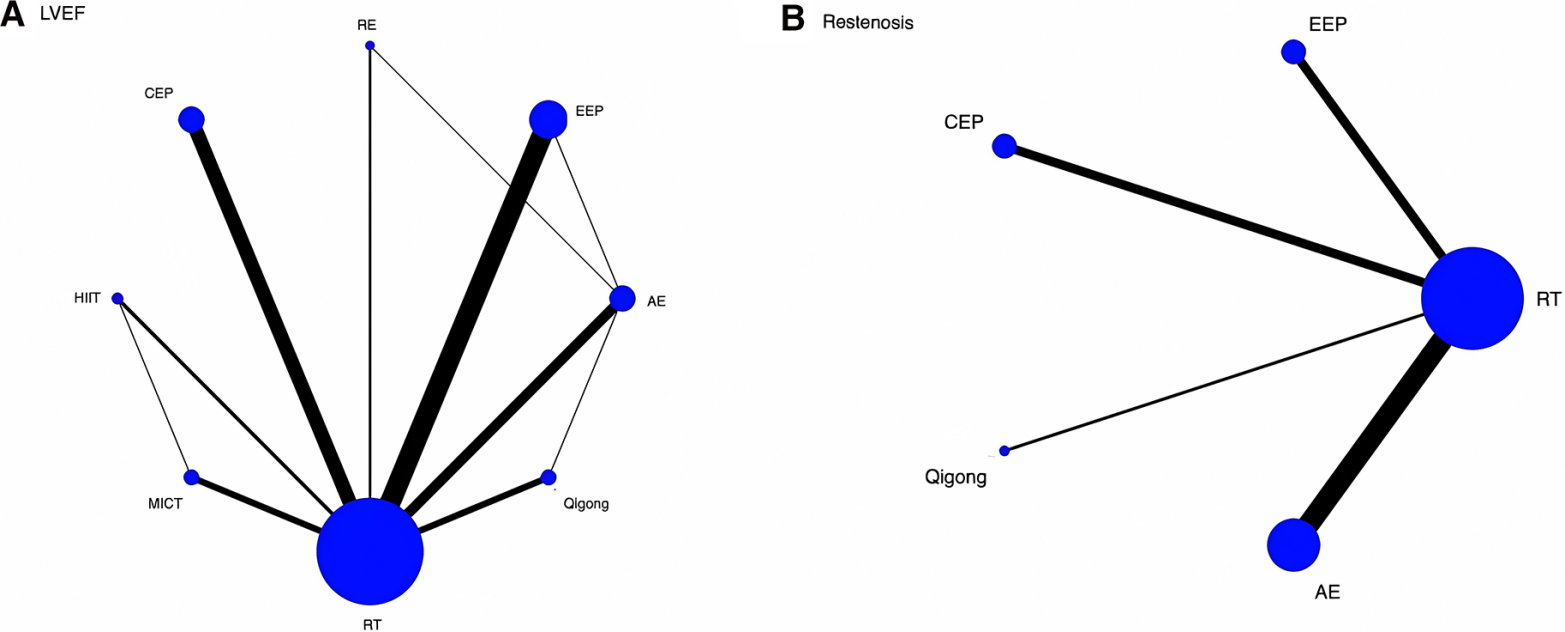
Network evidence plot.

The network evidence plot is shown in Figure 3 where the thicker the line between two (indicating the larger the number of studies compared between two), the larger the node and the larger the sample size of studies involved for this intervention type. (1) LVEF: The 57 included RCTs evaluated LVEF, forming a total of four closed loops **(3A)**, namely, RT-MICT-HIIT, RT-RE-AE, RT-EEP-AE, and RT-AE-Qigong, with the largest number of studies comparing EEP with RT, followed by RT with CEP. (2) Restenosis: Fourteen studies evaluated restenosis and did not form a closed loop **(3B)**, with most studies examining AE vs. RT and the fewest examining Qigong vs. RT.

#### 3.9.3. Comparison of the results of the net meta-analysis

**Figure 4:**
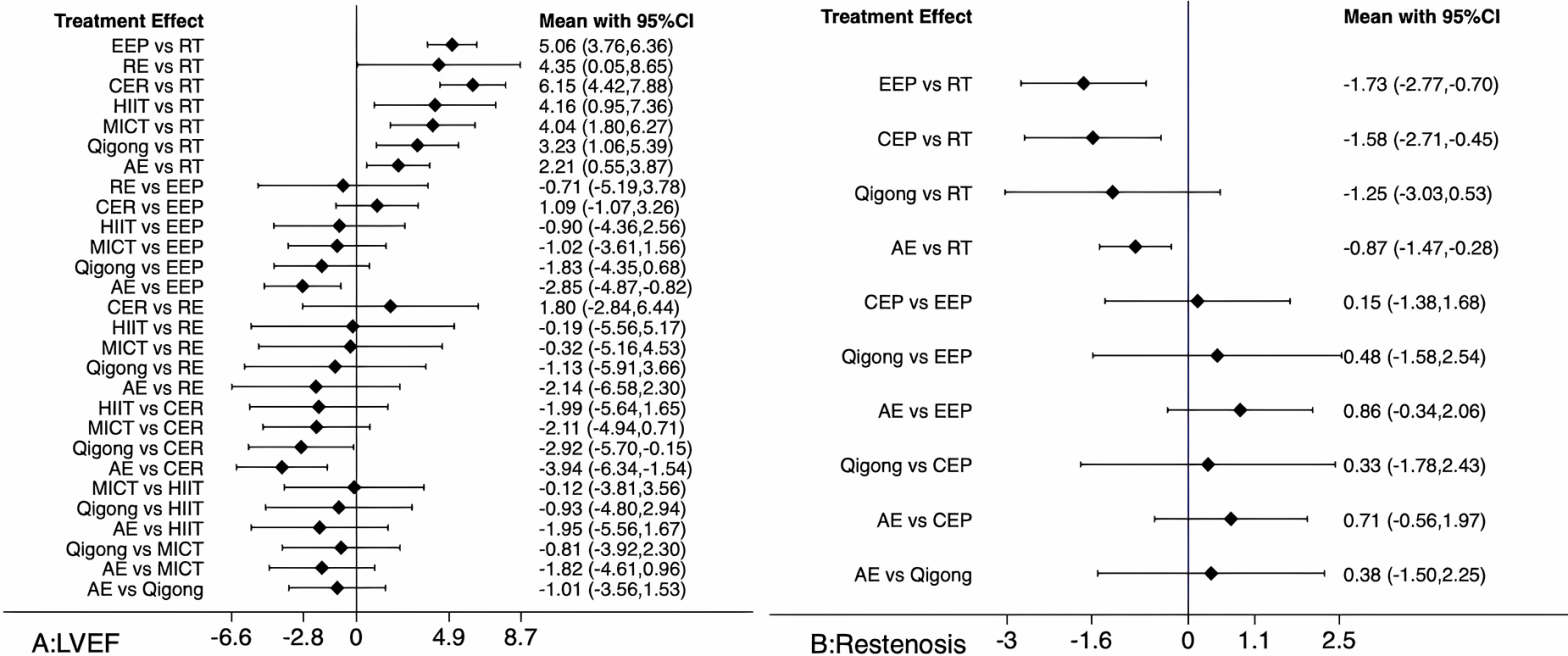
Network meta-analysis forest diagram.

The results of the NMA are shown in the forest plot (Figure 4): **4A** for LVEF and **4B** for restenosis. The two-by-two comparison between interventions in the forest plot, with the intersection with the 0 axis indicates that the difference between this group is not significant, and the specific values of graphs A and B are shown in Table 6 and Table 7, respectively.

(1) LVEF: All six ER protocols, RE, Qigong, MICT, HIIT, EEP, and AE, were superior to RT, and the differences were all significant; CEP vs. AE, ERP vs. AE, and HIIT vs. AE were all significant; and CEP, ERP, and HIIT were superior to AE.
(2) Restenosis: EEP, CEP, and AE were all superior to RT, with significant differences, while Qigong vs. RT was not significant.

**Table 6:** Network meta-analysis of the effect of different exercise modes on LVEF [MD (95% CI)].

RT							
−4.35 (−8.65, −0.05)^a^	RE						
−3.23 (−5.39, −1.06)^a^	1.13 (−3.66,5.91)	Qigong					
−4.04 (−6.27, −1.80)^a^	0.32 (−4.53,5.16)	−0.81 (−3.92,2.30)	MICT				
−4.16 (−7.36, −0.95)^a^	0.19 (−5.17,5.56)	−0.93 (−4.80,2.94)	−0.12 (−3.81,3.56)	HIIT			
−5.06 (−6.36, −3.76)^a^	−0.71 (−5.19,3.78)	−1.83 (−4.35,0.68)	−1.02 (−3.61,1.56)	−0.90 (−4.36,2.56)	EEP		
−6.15 (−7.88, −4.42)^a^	−1.80 (−6.44,2.84)	−2.92 (−5.70, −0.15)	−2.11 (−4.94,0.71)	−1.99 (−5.64,1.65)	−1.09 (−3.26,1.07)	CER	
−2.21 (−3.87, −0.55)^a^	2.14 (−2.30,6.58)	1.01 (−1.53,3.56)	1.82 (−0.96,4.61)	1.95 (−1.67,5.56)^a^	2.85 (0.82,4.87)^a^	3.94 (1.54,6.34)^a^	AE

SMD and 95% CI are >0 or <0 or indicate a significant difference between interventions (*p* < 0.05), marked with“^a^”.

**Table 7:** network meta-analysis of the effect of different exercise modes on restenosis [OR (95% CI)].

RT				
1.25 (−0.53,3.03)	Qigong			
1.73 (0.70,2.77)^a^	0.48 (−1.58,2.54)	EEP		
1.58 (0.45,2.71)^a^	0.33 (−1.78,2.43)	−0.15 (−1.68,1.38)	CEP	
0.87 (0.28,1.47)^a^	−0.38 (−2.25,1.50)	−0.86 (−2.06,0.34)	−0.71 (−1.97,0.56)	AE

SMD and 95% CI are >0 or <0 or indicate a significant difference between interventions (*p* < 0.05), marked with“^a^”.

#### 3.9.4. Ranking of quality measures

**Figure 5:**
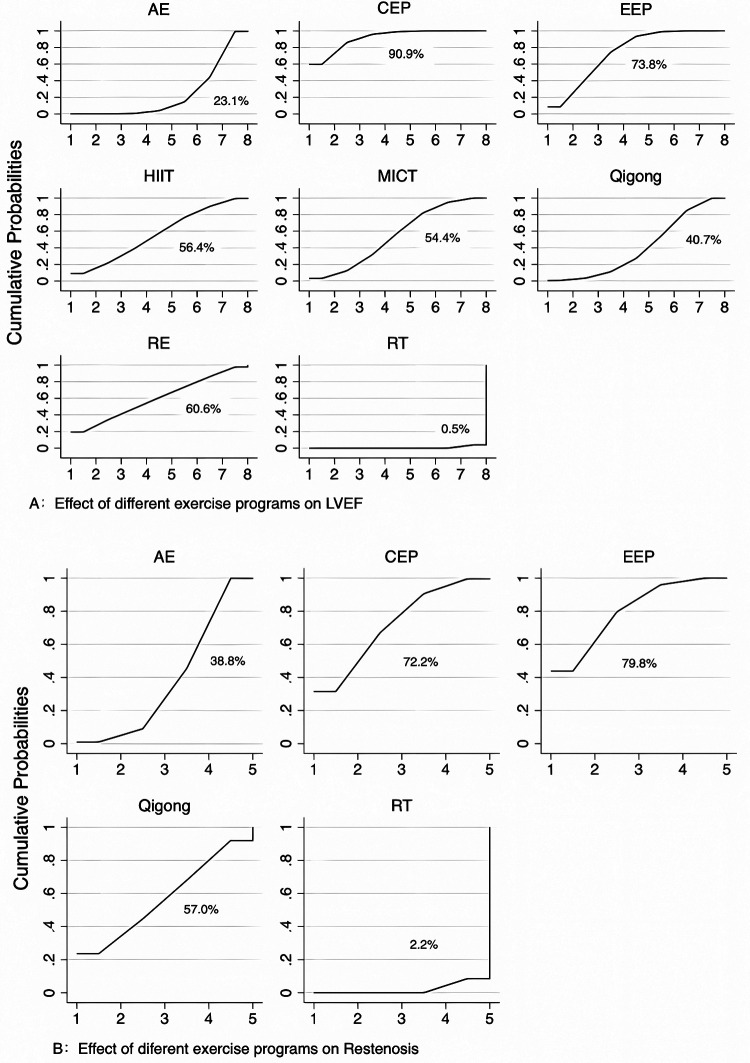
Sorting results of surfaces under the cumulative ranking curves.

The surface area under the effective cumulative curve represents the effectiveness of a particular intervention in improving the outcome indicators, with larger surface areas being more effective. The curves for LVEF and restenosis are shown in Figures A and B, respectively. The ranking results showed that CEP was most likely to improve LVEF in patients after PCI (90.9%), followed by RE, CEP, EEP, HIIT, Qigong, AE, and RT (Figure 5A). EEP most likely reduced the incidence of restenosis in patients after PCI, followed by CEP, Qigong, AE, and RT (Figure 5B).

#### 3.9.5. Publication bias analysis

**Figure 6:**
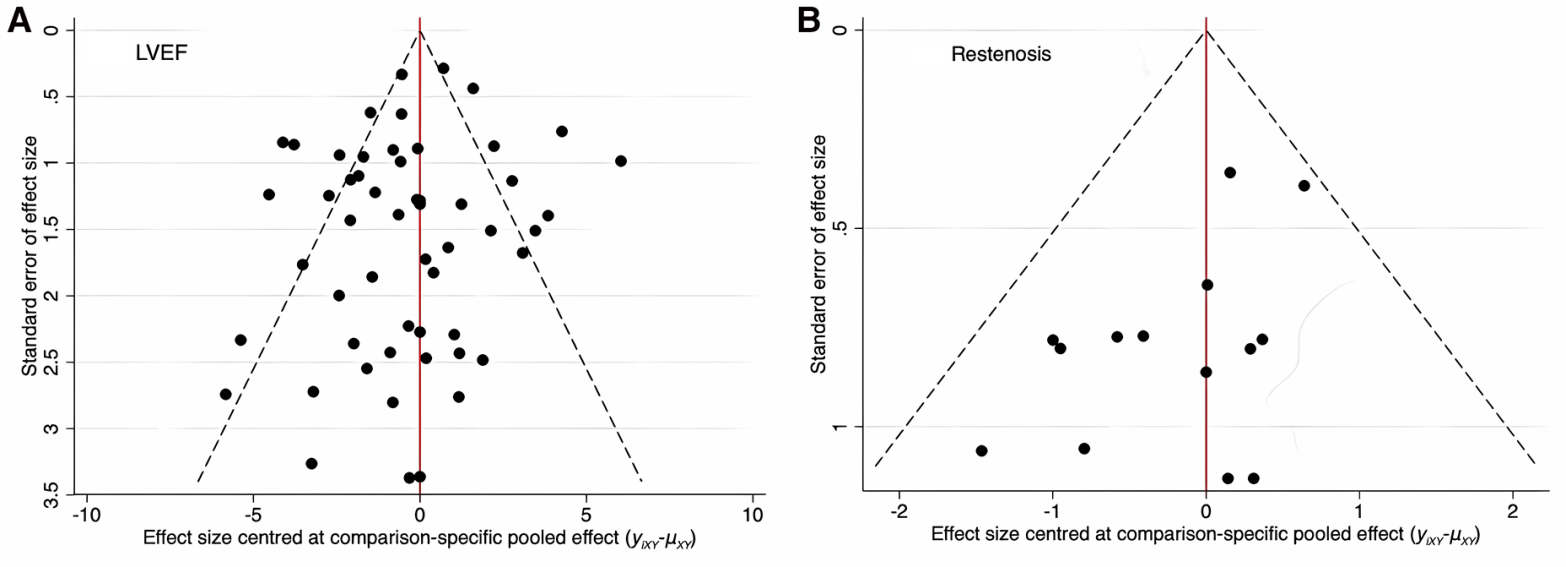
Comparison-adjusted funnel plot of the effective rates of different interventions.

There was little overlap between LVEF and restenosis in the reports of all RCTs included in this study; therefore, the two indicators were tested independently. Funnel plots are shown as Figures 6A and B. The more symmetrical the results, the less likely there was a publication bias analysis or a small sample effect.

LVEF: As shown in Figure 6A, three points fell outside the 95% CI, which may be related to the heterogeneity between studies. The funnel plot was symmetric, and the possibility of publication bias or small sample effect was small.

Restenosis: As shown in Figure 6B, all points were inside the funnel, but the point distribution was asymmetrical, and there may have been publication bias or small-sample effects.

## 4. Discussion

### 4.1. Quality evaluation of included meta-analyses

All included studies had very low methodological quality. The main reason involves significant shortcomings in two important aspects (2,7): none of the meta-analyses were preregistered, and a list of excluded literature was not provided. Other common problems include the following: (1) the authors did not investigate funding and conflicts of interest in the literature, (2) failure to characterize the included studies according to the PICOS principle, and (3) failure to discuss publication bias in the quantitative analysis results.

In terms of the report quality, we included only studies with relatively complete research reports. However, overall, there were serious deficiencies in the entry of “protocol and registration” and some deficiencies in the four items of “introduction rationale,” “eligibility criteria,” “search” and “limitations”: (1) all the studies were not registered in advance; (2) there was a lack of explanation of the review principle; (3) the status of the current research of the same type and the necessity of this research was lacking; (4) there was no reasonable explanation of the strategy; (5) only the search term is given, but the specific search strategy is not given; and (6) there was no statement of the limitations of the study.

As noted above, the deficiencies may be related to factors such as unregistered and unreviewed research design schemes, lack of awareness of report quality assessment tools during writing, and layout of limitations, which are common problems in current non-Cochrane analyses. This also shows that the writing of the included studies was not done strictly according to the checklist.

### 4.2. Qualitative analysis results

All meta-analyses evaluated ER as safe, and all high- and moderate-quality evidence levels of outcomes confirmed that ER is effective in improving cardiac output capacity and reducing the incidence of restenosis in patients after PCI, which is consistent with the current findings. A direct comparison of the amount of combined benefit found that early ER was significantly superior to other intervention options although this finding is not reliable due to possible overlap in the included literature between different meta-analyses.

### 4.3. Quantitative analysis results

First, we excluded RCTs with a high risk of bias from the screening process to increase confidence in the final analysis. The main reason for the high and medium risk of bias in RCTs of interest was the implementation of blinding and allocation concealment. Most of the included articles only mentioned blinding without describing the process; similarly, most of the articles only mentioned the use of a computerized random sequence for allocation without describing the people who performed this grouping. The majority of the included articles only mentioned the use of computerized random sequences for allocation but did not describe whether the participants in this grouping were informed of each other. Notably, male participants comprised the majority of subjects in the included RCTs, even more than six times the number of female participants in the studies, which is relevant to the population with CHD but also suggests that the results of such studies in the past may have been biased by sex and that future studies targeting women or comparing men with women are necessary. RCTs focusing on women or comparing men and women are necessary in the future.

Second, in the process of including research literature, we found that most researchers specified the type, frequency, and duration of exercise. However, except for protocols with defined exercise intensity, such as HIIT and MICT exercise and Chinese medicine Qigong, which has fixed exercise steps (46–48), other trainings mostly ignore the definition of exercise intensity in exercise protocols [Borg scales, maximum heart rate, and reserve heart rate are recommended to evaluate exercise intensity (49)]. There are also problems with the construction of EEP programs, which are mostly conducted by nurses, with a clear plan for the in-hospital training component but neglecting the out-of-hospital maintenance program. There is a lack of science in the design of ER programs, and thus, program development should be carried out jointly by physicians and kinesiologists.

The results of the reticulated meta-analysis indicate that all seven ER protocols (RE, Qigong, MICT, HIIT, EEP, CEP, and AE) are effective in improving LVEF in post-PCI patients, which is consistent with the qualitative analysis. The current results indicate that the CEP, ERP, and HIIT training protocols improve LVEF better than AE. It also shows that CEP has the highest potential to improve LVEF in post-PCI patients—which does not suggest that TCM Qigong reduces restenosis rates in post-PCI patients and which diverges from the previous qualitative analysis and may be a source of inconsistency in this set of analyses. However, the results of the publication bias analysis suggest the possibility of publication bias in restenosis studies, which may have an impact on the reliability of the results of this analysis.

### 4.4. Comparison of various ER options

#### 4.4.1. CEP vs. EEP

Critical CR organizations, such as AHA, AACVPR, CACR, and EACPR, all recommend a combined training approach. However, the implementation of CEP is mostly carried out in rehabilitation centers (relying on equipment and doctors), is more expensive, and may not be suitable for less developed countries and regions. Strictly speaking, EEP is similar to CEP in that it includes aerobic, resistance, and flexibility training, and the intervention is delivered earlier than CEP.

#### 4.4.2. HIIT, MICT, and RE

HIIT training and MICT training both have clear definitions and training modalities, and similar to RE, the implementation of HIIT and MICT is mostly device-dependent and physician-supervised. In the quantitative analysis of this study, we did not observe direct evidence that HIIT, MICT, and RE can reduce the incidence of reinfarction, but all three can improve LVEF, and more relevant high-quality future studies are needed. Compared to CEP, HIIT requires similar equipment, medical and nursing conditions, and less time to train, so it may be possible to participate in optimizing CEP protocols in the future.

#### 4.4.3. AE and qigong

AE and Qigong have the common advantages of being simple and easy to implement, not limited by space or equipment, and not imposing an additional financial burden on the patient, making them more suitable for community CR. AE has a low likelihood of improving both indicators, and CEP, ERP, and HIIT were superior to AE and were more time-consuming and less therapeutically meaningful. Although Qigong was superior to AE in improving LVEF and reducing the incidence of reinfarction in the likelihood comparison, the results of Qigong in reducing the incidence of reinfarction were not meaningful and could not be evaluated in this study. Thus, more studies need to be conducted in the future.

## 5. Conclusion

The methodological quality of the current meta-analysis examining the impact of ER on LVEF and coronary restenosis incidence in patients after PCI is low. This is mainly because there was no preregistration, no list of excluded literature was provided, and the writing of the included studies did not strictly follow the requirements of the checklist. The RCTs included were mostly unclear in their descriptions of blinding and allocation concealment and had a much larger proportion of male participants than female participants. The intervention methods included a comprehensive exercise program, early exercise program, high-intensity interval exercise, medium-intensity continuous training, resistance exercise, aerobic exercise, and Chinese Qigong. The results of the quantitative analysis showed that while all seven programs could improve LVEF, the comprehensive exercise program was the most likely to improve LVEF, and the comprehensive exercise program, early exercise program, and high-intensity interval exercise were better than aerobic exercise. Comprehensive exercise programs, early exercise programs, and aerobic exercise reduced the incidence of restenosis in patients. However, Chinese Qigong did not reduce the incidence of restenosis in patients, there was a risk of bias and inconsistency in the quantitative analysis of restenosis incidence, and more experimental evidence is needed in the future.

## Data Availability

The original contributions presented in the study are included in the article/**Supplementary Material**, further inquiries can be directed to the corresponding authors.
