# Supplementary material for: Effect of different exercise regimens on LVEF and restenosis incidence in patients after PCI: a network meta-analysis and an overview of systematic reviews
Source: Front Cardiovasc Med. 2023 Nov 14;10:1241343. doi: 10.3389/fcvm.2023.1241343 (PMC10686069; doi:10.3389/fcvm.2023.1241343)
Supplement: Supplementary file 2 [file Datasheet5.docx]

| **Supplemental Table1 :Characteristics of studies included in the network Meta-analysis** | | | | | | | | | | | |
| --- | --- | --- | --- | --- | --- | --- | --- | --- | --- | --- | --- |
| Rank | Study | **participant** | | | | **Interventions** | | **Outcome** | **Study** | | |
|  |  | Type | Number (E/C) | Age (E/C) | Sex (F/M) | E | C |  | Follow up | Type | Quality Assessment Level |
| 1 | Abtahi 2017 | After PCI | 25/25 | 53.76 ± 6.96/53.6 ± 6.98 | 27/23 | AE | RT | LVEF | 8W | RCT | B |
| 2 | Belardinelli 2001 | After PCI | 49/50 | 53.0±11.0/59.0±10.0 | 49/50 | CEP | RT | restenosis | 6W | RCT | B |
| 3 | Begigiene A 2021 | After PCI | 26/19 | 72.9±4.4/75.5±6.9 | 11/34 | RE | RT | LVEF Restenosis | 3W | RCT | A |
| 4 | Bai FF 2020 | After PCI | 125/125 | 52. 2 ± 5. 9/51. 3 ± 5. 2 | 129/122 | CEP | RT | LVEF | 6M | RCT | B |
| 5 | Belardinelli,2001 | After PCI | 59/59 | 53±11/59±10 | 99/19 | CEP | RT | LVEF | 6M | RCT | B |
| 6 | Cai HM 2020 | After PCI | 42/54 | 55.62±3.02/55.62±3.02 | 73/23 | EEP | RT | LVEF | 1w | RCT | B |
| 7 | Cai ZK 2017 | After PCI | 25/25 | 56.1±9.8 / 56.1±8.5 | 42/8 | EEP | RT | LVEF | 12w | RCT | B |
| 8 | Cheng PJ 2018 | After PCI | 30/30 | 57. 2 ± 6. 4/55. 4 ± 7. 1 | 42/18 | CEP | RT | LVEF | 6M | RCT | B |
| 9 | Dendale 2005 | After PCI | 149/83 | 62 ± 7/68 ± 8 | 161/71 | CEP | RT | Restenosis, | 6M | RCT | B |
| 10 | Dai RZ 2007 | After PCI | 33/34 | 60.19 ±9.61/62.33 ± 10.02 | 57/10 | EEP | RT | LVEF | 2w | RCT | B |
| 11 | Fan KL 2021 | After PCI | 42/42 | NP | NP | EEP | RT | LVEF | NP | RCT | B |
| 12 | Fan Z 2010 | After PCI | 23/24 | 62.0±5.6 | 39/8 | MICT | RT | LVEF | 6M | RCT | B |
| 13 | Francesco 2012 | After PCI | 25/21 | 54±7/ 54±9 | 40/6 | EEP | RT | LVEF | 14w | RCT | B |
| 14 | Francesco 2013 | After PCI | 24/26 | 54±7/ 52±10 | 46/4 | EEP | RT | LVEF | 6M | RCT | B |
| 15 | Fu YY 2017 | After PCI | 35/35 | 72. 35 ± 3. 84/73. 76 ± 4. 01 | 54/15 | AE | RT | LVEF | 6M | RCT | B |
| 16 | Gao Z 2015 | After PCI | 22/21/22 | 59.4± 7.9/61.2± 8.0/60.4± 8.6 | 51/14 | HIIT | RT/MICT | LVEF | 7W | RCT | B |
| 17 | Gao LM 2013 | After PCI | 47/47 | 58.12±8.45/23.08±2.36 | 53/41 | AE | RT | LVEF | 1Y | RCT | B |
| 18 | Gu SF 2018 | After PCI | 60/60 | 56.83±7.48/57.90±8.06 | 95/25 | EEP | RT | LVEF | 1W | RCT | B |
| 19 | Guo RR2018 | After PCI | 100 /100 | 60. 4±5. 4 / 60. 6±6. 3 | 105/95 | AE | RT | LVEF | 6M | RCT | B |
| 20 | Guo XY 2018 | After PCI | 52/52 | 36. 47 ± 6. 83/35. 73 ± 7. 42 | 41/63 | EEP | RT | LVEF | 6M | RCT | B |
| 21 | Gong H 2015 | After PCI | 100/100 | 59.0±7.1 | 160/40 | EEP | RT | Restenosis | 6M | RCT | B |
| 22 | Huan Zheng  2008 | After PCI | 27/30 | NP | NP | MICT | RT | LVEF | 7M | RCT | B |
| 23 | Huang S 2018 | After PCI | 28/28 | 59. 34 ± 14. 22/60. 9 ± 12. 81 | 37/19 | EEP | AE | LVEF | 6M | RCT | B |
| 24 | Jiang M 2021 | After PCI | 49/49 | 59.62±8.98/58.79±9.36 | 64/34 | RE | RT | LVEF | 6W | RCT | A |
| 25 | Jónsdóttir S 2006 | After PCI | 21/22 | 68.0±6.6/69.0±5.3 | NP | RE | AE | LVEF | 5W | RCT | A |
| 26 | Jiang QY 2017 | After PCI | 30/30 | 58. 44±5. 83 / 58. 82±5. 91 | 35/25 | AE | RT | LVEF | 3M | RCT | B |
| 27 | Juan Wang, 2020 | After PCI | 60/60 | 60.28 ± 2.82/59.36 ± 3.27 | 66/54 | CEP | RT | LVEF | 2W | RCT | B |
| 28 | Kim C 2015 | After PCI | 14/14 | 24.28± 2.93/ 24.64± 3.56 | 22/6 | HIIT | RT | LVEF | 6W | RCT | B |
| 29 | Kubo, 1992 | After PCI | 18/20 | 59±12/58±10 | 30/8 | MICT | RT | Restenosis | 12W | RCT | B |
| 30 | Koizumi, T 2003 | After PCI | 15/15 | 51±12/59±9 | 27/3 | AE | RT | LVEF | 3M | RCT | B |
| 31 | LiZC 2019 | After PCI | 40/40 | 55. 32 ± 9. 52/56. 47 ± 11. 83 | 57/23 | CEP | RT | LVEF | 3M | RCT | B |
| 32 | Liu YF 2005 | After PCI | 30/30 | 40 ± 10/49 ± 12 | 41/19 | Qigong | RT | LVEF | 3M | RCT | B |
| 33 | lee 2013 | After PCI | 37/37 | 58.8± 10.8 60.3±8.7 | 61/13 | EEP | RT | Restenosis | 9M | RCT | B |
| 34 | Li ML 2011 | After PCI | 74/74 | 65.1±5.7 | 79/69 | EEP | RT | Restenosis | 6M | RCT | B |
| 35 | Li MJ 2002 | After PCI | 25/18/20 | 57.1±12.3/56.4±11.5/57.5±11.8 | 57/6 | CEP | RT | Restenosis | 6M | RCT | B |
| 36 | LYM 2011 | After PCI | 42/48 | 69.7±10.1/65.9±9.2 | 61/29 | CEP | RT | Restenosis | 6M | RCT | B |
| 37 | LYM 2012 | After PCI | 50/52 | 69.7±10.8/70.1±11.9 | NP | CEP | RT | Restenosis | 6M | RCT | B |
| 38 | Liu D 2023 | After PCI | 42/42 | 64.08±4.14/64.78±4.21 | 46/39 | EEP | RT | LVEF | 12W | RCT | B |
| 39 | Mei 2009 | After PCI | Not mentioned | 64.0±9.1/ 64.0±9.1 | Not mentioned | AE | RT | Restenosis | 6M | RCT | B |
| 40 | Ming-Gui Chen  2020 | After PCI | 48/48 | 59.36 ± 3.27/59.98 ± 10.86 | 42/41 | Qigong | RT | LVEF | 6M | RCT | B |
| 41 | Ma J 2023 | After PCI | 40/40 | 62.85±5.92/61.85±5.92 | 52/28 | EEP | RT | LVEF | 1M | RCT | B |
| 42 | Munk P 2009 | After PCI | 20/18 | 57.7± 10.4/ 59.7± 8.5 | 32/6 | HIIT | RT | LVEF | 6M | RCT | B |
| 43 | Mei J, 2009 | After PCI | 150/150 | 67.0±9.1 | NP | EEP | RT | Restenosis | 6M | RCT | B |
| 44 | Niu F 2017 | After PCI | 132/124 | 62. 63 ± 2. 23/62. 32 ± 2. 53 | 136/120 | AE | RT | LVEF | 6M | RCT | B |
| 45 | Qu LN 2017 | After PCI | 54/54 | NP | NP | AE | RT | LVEF | 1M | RCT | B |
| 46 | Song YL 2018 | After PCI | 44/52 | 60. 78±9. 03 / 58. 38±8. 37 | 50/46 | Qigong | RT | LVEF | 3M | RCT | B |
| 47 | Sheng W 2003 | After PCI | 17/15 | 57 ± 13/55 ± 14 | 26/6 | CEP | RT | LVEF | 3M | RCT | B |
| 48 | Tian ZW 2016 | After PCI | 30/30 | 61. 4 ± 6. 9/60. 5 ± 6. 5 | 50/10 | AE | RT | LVEF | 3M | RCT | B |
| 49 | Vasiliauskas,  2007 | After PCI | 95/90 | 58.4±4.3/ 59.5±5.2 | NP | EEP | RT | LVEF Restenosis | 6M | RCT | B |
| 50 | Wang AL 2021 | After PCI | 40/40 | 58.91 ± 6.72/58.72 ± 6.23 | 46/ 34 | EEP | RT | LVEF | 1W | RCT | B |
| 51 | Wang Y, 2016 | After PCI | 29/29 | 55.28±3.10 / 54.14±4.87 | 39/19 | AE | RT | LVEF | 6M | RCT | B |
| 52 | Wang JJ 2019 | After PCI | 55/55 | 58.32±9.74/60.32±7.23 | 55/55 | Qigong | RT | LVEF | 5M | RCT | B |
| 53 | Wang JM 2018 | After PCI | 75 /75 | 59. 3±15. 4 / 58. 8±12. 5 | 108/42 | Qigong | AE | LVEF | 6M | RCT | B |
| 54 | Wu.2013 | 30/30 | 37/36 | 68.2±12.04/ 68.3±10.9 | 37/36 | CEP | RT | Restenosis | 6M | RCT | B |
| 55 | WU XB 2016 | After PCI | 38/38 | 50 ± 10/49 ± 8 | 45/31 | CEP | RT | LVEF | 4M | RCT | B |
| 56 | Xue AL 2018 | After PCI | 25/25 | 57.24±9.13/58.40±9.57 | 45/5 | EEP | RT | LVEF | 1w | RCT | B |
| 57 | Xu 2017 | After PCI | 58/60 | 56.40±8.12/ 58.62±7.96 | 93/15 | CEP | RT | LVEF | 6M | RCT | B |
| 58 | Xu L 2016 | After PCI | 26/26 | 55.8 ± 9.7/ 55.5 ± 8.9 | 47/9 | EEP | RT | LVEF | 6M | RCT | B |
| 59 | Yong Zhang  2018 | After PCI | 65/65 | 70.3 ± 10.7/60.28 ± 2.82 y | 103/27 | MICT | RT | LVEF | 4M | RCT | B |
| 60 | Yang GH 2019 | After PCI | 47/51 | 55. 2 ± 10. 1/60. 2 ± 10. 4 | 83/15 | CEP | RT | LVEF | 3M | RCT | B |
| 61 | YU HM 2018 | After PCI | 75/75 | 58. 27 ± 4. 15/59. 53 ± 3. 86 | 97/53 | AE | RT | LVEF | 6M | RCT | B |
| 62 | Zhang ZL 2019 | After PCI | 33/34 | 59.42±7.022/58.65±7.027 | 44/23 | Qigong | RT | LVEF | 8W | RCT | B |
| 63 | Zhang H2015 | After PCI | 40/40 | 60. 25±8. 35 / 58. 08±6. 08 | 44/35 | AE | RT | LVEF | 3M | RCT | B |
| 64 | Zhou YL2013 | After PCI | 51 /51 | 53±5 /53±5 | NP | Qigong | RT | LVEF | 3M | RCT | B |
| 65 | Zhao DJ 2020 | After PCI | 40/40 | 58. 4 ± 10. 1/57. 4 ± 9. 4 | 69/11 | AE | RT | LVEF | 3M | RCT | B |
| 66 | Zheng XW 2019 | After PCI | 46/46 | 60. 25 ± 10. 21/59. 82 ± 10. 02 | 53/27 | CEP | RT | LVEF | 3M | RCT | B |
| 67 | Zhou DL 2016 | After PCI | 24/20 | 60. 82 ± 8. 37/61. 6 ± 8. 71 | 29/13 | AE | RT | LVEF | 6M | RCT | B |
| 68 | Zhang XF 2018 | After PCI | 50/50 | 57. 19 ± 7. 54/56. 71 ± 7. 68 | 68/32 | Qigong | RT | LVEF | 6M | RCT | B |
| 69 | Zhang BH 2006 | After PCI | 21/18 | NP | 35/4 | MICT | RT | Restenosis, | 6M | RCT | B |
| 70 | Zhou XH 2018 | After PCI | 42/42 | 49±6. 65 / 51±7.49 | 44/40 | EEP | RT | LVEF | 1W | RCT | B |
| Note:E:experimental group; C: control group; W: week; M: month RCT: randomised controlled trial PCI: percutaneous coronary intervention; AE: aerobic exercise; RT: routine treatment; EEP: early exercise programme; RE: resistance exercise; CEP: combined exercise programme; HIIT: high-intensity interval exercise; MICT: moderate-intensity continuous training; Qigong: Chinese medicine qigong.NP:None reportedZhang | | | | | | | | | | | |

[1]Abtahi, F.; Tahamtan, M.; Homayouni, K.; Moaref, A.; Zamirian, M. Assessment of Cardiac Rehabilitation on Echocardiographic Parameters of Left Ventricular Systolic Function in Patients Treated by Primary Percutaneous Coronary Intervention due to Acute ST-Segment Elevation Myocardial Infarction: A Randomized Clinical Trial. Int. Cardiovasc. Res. J. 2017, 11, 130–136.

[2] Belardinelli R, Paolini I, Cianci G, Georgiou D al.: Exercise training intervention after coronary angioplasty: the ETICA trial. J Am Coll Cardiol 2001; 37 (7): 1891-1900. doi: 10.1016/s0735-1097(01)01236-0.

[3] Beigienė A, Petruševičienė D, Barasaitė V, Kubilius R, Macijauskienė J. Cardiac Rehabilitation and Complementary Physical Training in Elderly Patients after Acute Coronary Syndrome: A Pilot Study. Medicina (Kaunas) 2021; 57 (6): doi: 10.3390/medicina57060529.

[4] BAI Fangfang, Lijun ZHU, Yongmei KONG, et al. Benefits of cardiac rehabilitation in patients after percutaneous coronary intervention for coronary artery disease [J]. Journal of Integrative Cardiovascular Diseases, 2020, 18( 3) : 466 469.(in chinese)

[5] Belardinelli R, Paolini I, Cianci G, Georgiou D al.: Exercise training intervention after coronary angioplasty: the ETICA trial. J Am Coll Cardiol 2001; 37 (7): 1891-1900. doi: 10.1016/s0735-1097(01)01236-0.

[6] Huimin Cai. Early rehabilitation program for acute myocardial infarction undergoing emergency PCI [J]. Evidence-based Nursing, 2020, 6(10): 1106.(in chinese)

[7] Cai Zekun, Xu Lin, Ma Jun, et al. The effect of early cardiac rehabilitation procedures on cardiac systolic function after percutaneous coronary intervention in patients with acute myocardial infarction [J]. Chinese Journal of Rehabilitation Medicine, 2017, 32(4):391-395.(in chinese)

[8] Chen Peijin, Liu Yingxia, Sun Huafeng, et al. Application of phase II cardiac rehabilitation therapy to patients with acute myocardial infarction [J]. Guangdong Medicine, 2018, 39 ( 7) :1017 1021.(in chinese)

[9] Dendale P, Berger J, Hansen D, Benit E al.. Cardiac rehabilitation reduces the rate of major adverse cardiac events after percutaneous coronary intervention. Eur J Cardiovasc Nurs 2005; 4 (2): 113-116. doi: 10.1016/j.ejcnurse.2004.11.003.

[10] Dai Ruozhu, Chen Tianbao, Wang Ling et al. Effect of cardiac procedural rehabilitation on the outcomes of patients undergoing emergency percutaneous coronary intervention [J]. Journal of Cardiovascular Rehabilitation Medicine, 2007, 16(5):426.(in chinese)

[11] Fan Kaili, Yang Huiyun, Wu Yanni, et al. Clinical effect of Phase I cardiac rehabilitation exercise on patients after percutaneous coronary intervention for acute myocardial infarction [J]. Chinese Journal of Practical Nursing, 2021, 37(18),1394.(in chinese)

[12] Fan, Z., Sun P., Zhang J., Li Y. Effect of exercise training on oxygen metabolic equivalent and left ventricular function in patients.

for acute myocardial infarction after PCI intervention J. Am. Coll. Cardiol. 2015, 66, C234.

[13] Giallauria F, Acampa W, Ricci F, Vitelli A, Torella G, Lucci R, et al. Exercise training early after acute myocardial infarction reduces stress-induced hypoperfusion and improves left ventricular function. Eur J Nucl Med Mol Imaging 2013; 40 (3): 315-324. doi: 10.1007/s00259-012-2302-x.

[14] Giallauria F, Acampa W, Ricci F, Vitelli A, Maresca L, Mancini M, et al. Effects of exercise training started within 2 weeks after acute myocardial infarction on myocardial perfusion and left ventricular function: a gated SPECT imaging study. Eur J Prev Cardiol 2012; 19 (6): 1410-1419. doi: 10.1177/1741826711425427.

[15] Fu Yang Yang, Gao Chun Hong. Effect of systematic cardiac rehabilitation care on acute myocardial infarction Cardiac Function and Prognosis after Percutaneous Coronary Intervention in Patients [J]. Integrating Chinese and Western Medicine Nursing (Chinese and English) 2017, 3( 11):24 27.

[16] Gao, Z. Z., Ji, P., Xia, Y. Q., & Wang, L. (2015). Effects of different aerobic exercise intensities on cardiac function and exercise endurance patients after percutaneous coronary intervention. Chinese Journal of Rehabilitation Medicine, 30, 344–348.

[17] Gao Lame, Zhu Jianli. Effects of aerobic exercise on cardiopulmonary function, exercise capacity, and quality of life in patients with coronary heart disease after PCI[J]. Chinese and Foreign Medical Research,2023,21(08):145-149. DOI:10.14033/j.cnki.cfmr.2023.08.036. (In chinese)

[18] Gu Shufang, Yu Yanyan, and Zhang Limin. Effect of immediate rehabilitation training after coronary intervention in patients with acute myocardial infarction [J]. The Chinese Journal of Nursing 2018, 53.

(2): 173-178.(In chinese)

[19] Guo Ruirui, Chen Junmin, and Wang Ji. Analysis of the effect of cardiac rehabilitation training after percutaneous coronary intervention in patients with acute myocardial infarction [J]. Journal of Preventive Medicine of the PLA, 2018 ( 9) : 1108-10.

Guo Xueying

[20]. Effect of resistance training combined with aerobic exercise at 8,000 brisk steps on the rehabilitation of young patients: The effect of resistance training combined with 8000 steps of aerobic exercise on the recovery of young patients after PCI for coronary artery disease [J]. Modern Clinical Nursing, 2018, 17( 12) : 25 30.

[21] Gong Hong, Liu Ting, and Liu Yujuan. Effect of early rehabilitation care on the outcome of post-stent restenosis in coronary artery disease Effect of early rehabilitation care on the outcome of post-stenting restenosis in coronary artery disease. (in chinese)

[22] Zheng H, Luo M, Shen Y, Ma Y, Kang W. Effects of 6 months exercise training on ventricular remodelling and autonomic tone in patients with acute myocardial infarction and percutaneous coronary intervention. J Rehabil Med 2008; 40 (9): 776-779. doi: 10.2340/16501977-0254.

[23] Huang Sen, Su Mai, Su Hong, et al. The effect of exercise on cardiac rehabilitation after PCI in patients with coronary artery disease [J]. Journal of Integrative Cardiovascular and Cerebrovascular Diseases, 2018, 16( 20):3004 3007.(in chinese)

[24] Jiang M, Hua M, Zhang X, Qu L, Chen L. Effect analysis of kinetic energy progressive exercise in patients with acute myocardial infarction after percutaneous coronary intervention: A randomized trial. Ann Palliat Med 2021; 10 (7): 7823-7831. doi: 10.21037/apm-21-1478.

[25] Jónsdóttir S, Andersen KK, Sigurosson AF, et al.. Effect of physical training on chronic heart failure. Eur J Heart Fail 2006; 8 (1): 97-101. doi: 10.1016/j.ejheart.2005.05.002.

[26] Jiang QY, Lu M, Li JY et al. Analysis of the efficacy of cardiac rehabilitation therapy in patients with acute myocardial infarction after PCI [J]. Chinese and Western Medical Journal of Combined Cardiovascular and Cerebrovascular Diseases, 2017, 15(9):1036-1038.(in chinese)

[27] Wang, J. Clinical efficacy of early cardiac rehabilitation nursing for patients with acute myocardial infarction after interventional therapy. Int. J. Clin. Exp. Med. 2020, 13, 7986–7992.

[28] Kim C, Choi HE and Lim MH. Effect of high interval training in acute myocardial infarction patients with drug-eluting stents Am J Phys Med Rehabil 2015; 94 (10 Suppl 1):879-886. doi: 10.1097/phm.0000000000000290.

[29] Kubo H, Yano K, Hirai H et al.: Preventive effect of exercise training on recurrent stenosis after percutaneous transluminal coronary angioplasty (PTCA). Jpn Circ J 1992; 56 (5): 413-421. doi: 10.1253/jcj.56.413.

[30] Koizumi T, Miyazaki A, Komiyama N, Sun K, Nakasato T, Masuda Y, et al. Walking improves left ventricular dysfunction during exercise in patients with successful percutaneous coronary intervention for acute myocardial infarction. Circ J 2003; 67 (3): 233-237. doi: 10.1253/circj.67.233.

[31] Li Zhengchun. The value of phase II cardiac rehabilitation after PCI in patients with acute myocardial infarction [J]. Chinese convalescent medicine, 2019, 28 ( 2):151–153.(in chinese)

[32] Liu Yifu, Feng Xuemei. The effect of rehabilitation therapy on the efficacy of interventional treatment for coronary heart disease: impact of rehabilitation on the outcome of interventional treatment for coronary heart disease [J]. Journal of Continuing Medical Education, 2005, 28( 7):38 39.(in chinese)

[33]Lee HY, Kim JH, Kim BO, Byun YS, Cho S, Goh CW, et al. Regular exercise training reduces coronary restenosis after percutaneous coronary intervention in patients with acute myocardial infarction. Int J Cardiol 2013; 167 (6): 2617-2622. doi: 10.1016/j.ijcard.2012.06.122.

[34] Effect of nursing intervention on rehabilitation and prevention of restenosis after PTCA stenting for acute myocardial infarction [J]. China Modern Drug Application, 2011, 5 ( 2):205 - 206.(in chinese)

[35] Li M. J., Jiang H., Liu C.. Effect of rehabilitation exercise therapy on collateral circulation and restenosis after coronary angioplasty [J]. Chinese Journal of Rehabilitation Medicine, 2002, 17( 2):90–92.(in chinese)

[36]The effect of exercise training on the restenosis rate of large-diameter coronary stents. The effect of exercise training on the restenosis rate of large diameter coronary stents Journal of Vascular Rehabilitation Medicine, 2011, 20(5):416 - 419. (in chinese)

[37] Lu Yuming, Mao Hua, Tang Yan, et al. Effectiveness of post-PCI rehabilitation in elderly patients with coronary artery disease. Journal of Vascular Rehabilitation Medicine, 2 0 1 2 1 (6):5 8 6 - 5 8 8. (in chinese)

[38] Liu D. Effect of resistance training with aerobic exercise program on the rehabilitation effect of patients after PCI for myocardial infarction[J]. Jilin Med,2023,44(02):518-520.(in chinese)

[39] Mei, et al. Effects of early exercise prescription on myocardial infarction Patients rehabilitation and restenosis after stenting

Implantation plus Percutaneous Transluminal Coronary Angioplasty. Nurs J Chin PLA. 26, 11–13 (2009).

[40] Chen MG, Liang X, Kong L, Wang J, Wang F, Hu X, et al. Effect of Baduanjin Sequential Therapy on the Quality of Life and Cardiac Function in Patients with AMI After PCI: A Randomized Controlled Trial. Evid Based Complement Alternat Med 2020; 2020 8171549. doi: 10.1155/2020/8171549.

[41] Ma Juan,Wang Dongwei. Effect of individualized precision exercise as the core rehabilitation program on functional recovery of patients after coronary heart disease PCI[J]. Clinical Research,2023,31(05):85-88.(in chinese)

[42] Munk PS, Butt N, Larsen AI. High-intensity interval exercise training improves heart rate variability in patients following percutaneous coronary intervention for angina pectoris. Int J Cardiol 2010; 145 (2): 312-314. doi: 10.1016/j.ijcard.2009.11.015.

[43] Mei, J., Guo X., Sun J., Qi S., Yang L, Lei, S. (2009) Effects of early exercise prescription on acute myocardial infarction patients ' rehabilitation and restenosis after stent implantation. plus Percutaneous Transluminal Coronary Angiogplasty. Nursing Journal of Chinese PLA 26, 11-13.

[44] Niu F and Wang JY. Effect of exercise rehabilitation on cardiac function and major adverse events in patients undergoing percutaneous transluminal coronary intervention The effect of exercise rehabilitation on cardiac function and the incidence of major adverse cardiac events in patients undergoing percutaneous transluminal coronary intervention [J]. Journal of Practical Medicine, 2017, 34( 2):110 112.

[45] Dui LN, Cui YF, Yu B. Effect of early rehabilitation on left ventricular ejection fraction and exercise tolerance in patients with acute heart attack undergoing emergency PCI via the radial artery [J]. Journal of Practical Clinical medicine, 2017, 21(8):5.(in chinese)

[46] Sun Yangli, Sun Bingbing, Li Zhengyan et al.. Effect of cardiac rehabilitation intervention on left ventricular ejection fraction and exercise tolerance in patients with coronary artery disease after PCI [J]. Guangdong Medicine 2018 ( 24):1-4.(in chinese)

[47] Shen Wei, Li Tingfu. Analysis of the effects of interventional rehabilitation in patients after percutaneous transluminal coronary angioplasty and stenting [J]. Analysis of the effects of interventional rehabilitation in patients after percutaneous intracoronary angioplasty and stenting [J]. Chinese Clinical Rehabilitation, 2003, 7( 24):3287.(in chinese)

[48] Tian Zhaowei, Xu Ruqin, Wang Li, et al. Effect of guided home exercise on stage III cardiac rehabilitation in patients after coronary artery stenting [J]. Guangdong Medicine, 2016, 37( 16) : 2402 2405.(in chinese)

[49] Vasiliauskas D, Benetis R, Jasiukeviciene L, Grizas V, Marcinkeviciene J, Navickas R, et al. Exercise training after coronary angioplasty improves cardiorespiratory function. Scand Cardiovasc J 2007; 41 (3):142-148. doi: 10.1080/14017430601187116.

[50] Wang Qiaoli, Yujiao Wang. Effectiveness of early rehabilitation exercise instructions in patients after PCI for acute heart attack [J]. Thrombosis and Hemostasis, 2021, 27(1):14(in Chinese).

[51] Wang, Y. (2016) Effects of training on cardiac function and quality of life in CHD patients treated with PCI. Jilin University Journal 1-39.

[52] Wang JJ, Li RZ, and Yue LY. Application of seated eight-danjin exercises in the rehabilitation care of patients with coronary artery disease after PCI[J]. Harbin Medicine, 2019, 39(5): 465-467.(in chinese)

[53] Wang Jiamei, Liang Chun, Wang Bei, et al.. Effect of Eight Duan Jin sitting on post-intervention cardiac rehabilitation in patients with acute myocardial infarction [J]. Journal of Integrative Medicine and Cardiovascular Diseases, 2018; 16( 8):1082-5.(in chinese)

[54] Wu, X. H., Su, Y. Y., Zhang, C. X. & Mao, X. Q. Effect of Rehabilitation Therapy on Cardiovascular Events and Quality of Life in Patients with Coronary Heart Disease undergoing PCI. China’s Healthcare Nutrition. 9, 92–93 (2013).

[55] Wu Jinbo, Ye Xiaohan, Dong Mingguo, et al. Effect of rehabilitation therapy on cardiac function and quality of life in patients with sTable angina pectoris after intervention [J]. Journal of Heart, 2016, 28( 3) : 330 334.(in chinese)

[56] Xue A-L, Yuan B, Yuan Y-H et al. Evaluation of the efficacy of in-hospital early cardiac rehabilitation after emergency percutaneous coronary intervention in patients with acute ST-segment elevation myocardial infarction [J]. Chinese Journal of Hemorheology, 2018, 28(1): 31.(in chinese)

[57] Xu, Y., Feng Y., Su P., Li C., Qiao J. (2017) Impact of exercise rehabilitation on cardiac function in patients with coronary artery disease after percutaneous coronary intervention. Chinse

Circulation Journal 32, 326-330.

[58] Xu L, Cai Z, Xiong M, Li Y, Li G, Deng Y, et al. Efficacy of an early home-based cardiac rehabilitation program for patients after acute myocardial infarction: A three-dimensional speckle-tracking echocardiography randomized trial. Medicine (Baltimore) 2016; 95 (52): e5638. doi: 10.1097/md.0000000000005638.

[59] Zhang Y, Cao H, Jiang P, Tang H. Cardiac rehabilitation in acute myocardial infarction patients after percutaneous coronary intervention: A community-based study. Medicine (Baltimore) 2018; 97 (8): e9785. doi: 10.1097/md.0000000000009785.

[60] Yang Guohui, Shuren Li, Xuan Liu, et al. Study on the effect of home-directed cardiac exercise rehabilitation on patients with revascularized acute myocardial infarction after emergency percutaneous coronary intervention [J]. Journal of Practical Cardiopulmonary Vascular Disease, 2019, 27( 1) : 9 13, 18.(in chinese)

[61] Yu Hongmei: The effect of exercise training on the rehabilitation of patients after percutaneous coronary artery intervention [J]. Effect of exercise training on rehabilitation of patients after percutaneous coronary intervention [J]. Journal of Cardiovascular Rehabilitation Medicine, 2018, 27( 6) :637 641.(in chinese)

[62] Zhang Zhanglu. Evaluation of the efficacy of Ba Duan Jin in phase II cardiac rehabilitation of patients after emergency PCI [D]. Fuzhou: Fujian University of Traditional Chinese Medicine, 2019.(in chinese)

[63] Zhang Hui. Clinical observation of post-PCI rehabilitation treatment in patients with coronary heart disease [D]. Xinxiang Medical College, 2015.(in chinese)

[64] Zhou Yali . Effect of exercise therapy on exercise capacity in patients with coronary artery disease after interventional therapy [J]. Journal of Integrative Medicine and Cardiovascular Diseases, 2013; 11( 11):1389-90.(in chinese)

[65] Zhao Dongjing, Tang Wei, Cao Shujun, et al. Application of in-hospital + home continuous cardiac rehabilitation model in patients with acute myocardial infarction after emergency coronary intervention [J]. Chinese general medicine, 2020, 23 ( 16):73 78.

[66]Zheng Xiawen. Effect of elastic band resistance exercise on exercise capacity and rehabilitation process of coronary heart disease patients after PCI [J]. The effect of elastic band resistance exercise on exercise capacity and rehabilitation process of patients with coronary artery disease [J]. Chinese and Foreign Medical Research, 2019, 17 ( 23) : 166 168.

[67] Zhou DAL, Yu XY, Wei L, et al. Study on the improvement of prognosis of patients with acute myocardial infarction by aerobic exercise [J]. Chinese Journal of Geriatric Cardiovascular Diseases, 2016, 18( 7) : 702 705.(in chinese)

[68] Zhang Xiaofang, Jin Yu, Wu Yafang. Effect of rehabilitation training based on timing theory on cardiac function and quality of life in patients with unsTable angina undergoing PCI [J]. Chinese general medicine, 2018, 16( 4) : 671 674.(in chinese)

[69]Zhang Baohui, Li Xiaowen, Wang Xiaohong. Effect of regular exercise and physical activity on coronary artery restenosis after PTCA for coronary artery disease [J]. Journal of Cardiovascular Rehabilitation Medicine, 2006, 15( 1) : 130 - 132.(in chinese)

[70] Zhou Xiuhong, Du Xinping, Li Wenjie, et al. Study on the nursing care of early rehabilitation exercise training in patients with acute myocardial infarction after thrombus aspiration [J]. Journal of Nurse Training, 2018, 33(10):7-10, 19.
